# Alarm Therapy in the Treatment of Enuresis in Children: Types and Efficacy Review

**DOI:** 10.7759/cureus.17358

**Published:** 2021-08-22

**Authors:** Edris M Alqannad, Abdullah S Alharbi, Raghad A Almansour, Mohammed S Alghamdi

**Affiliations:** 1 General Practice, Ministry of Health, Makkah, SAU; 2 Family Medicine, Armed Forces Medical Center, Makkah, SAU; 3 Faculty of Medicine, Imam Mohammed ibn Saud University, Riyadh, SAU; 4 Faculty of Medicine, Albaha University, Albaha, SAU

**Keywords:** enuresis, nocturnal enuresis, alarm, efficacy, review

## Abstract

Enuresis is defined as bedwetting in children aged five years and older when organic reasons have been ruled out. It can result in substantial psychological repercussions and uncomfortable circumstances for both the child and the family. Medical (desmopressin, tricyclic antidepressants [TCAs]) and behavioral treatment are the basis for the treatment of enuresis. Alarm therapy is considered the first treatment modality of choice for enuresis with almost 50% cure rates are in the long term. Cooperation and compliance from parents and children are the cornerstones of the effectiveness of alarm therapy. Multiple factors, such as technical issues, might slow down the therapeutic response time. The objective of this study is to review the role of alarm therapy in the treatment of enuresis, its types, and its efficacy and to explore the factors that may increase or decrease its efficacy.

## Introduction and background

The most frequent type of urine incontinence in children is nocturnal enuresis, often known as bedwetting. Enuresis is defined by the World Health Organization (WHO) International Classification of Diseases 10th Revision (ICD-10) as bedwetting in children aged five years and older when organic reasons have been ruled out [1-2].

Despite the fact that enuresis is a discomfort issue that poses no harm to one's physical health, both the child and the family experience substantial psychological repercussions, causing stress and uncomfortable circumstances as a result of it. Also, it may have a detrimental impact on the development of self-confidence and the social skills of the child, as well as an association with poorer performance in school. It is classified as "primary" if no previous continence has been attained or "secondary" if at least six months of dry nights have been obtained [2-4].

Among children at age five, about 15% have nocturnal enuresis and 15% of them resolve spontaneously annually [3]. 

Enuresis is approximately two times more common in boys than girls, and it becomes less common with aging. According to a standard textbook, the prevalence is 7% for males and 3% for females at age five, 3% for males and 2% for females at age 10, and 1% for males and extremely rare in females at age 18 [1].

Enuresis has multiple etiologies ranging from bladder hyperactivity, waking up difficulties when the bladder is full, and increased nocturnal urine output. The genetic cause also cannot be ignored [3].

Because of the multiple and unclear factors leading to child enuresis, the treatment of enuresis is challenging for physicians and needs to be adjusted and individualized. The treatment of patients depends on the risk and benefit ratio and the therapeutic intervention effectiveness [3].

## Review

Treatment in general

Medical (desmopressin, tricyclic antidepressants [TCAs]) and behavioral treatment are the basis of enuresis treatment and should be started from age six in any child who has at least one enuresis episode per week. These modalities of treatment can be used alone or in combination. The gold standard treatment for enuresis is the behavioral night alarm [3,5].

Alarm

The alarm device consists of a sound system linked to a humidity detector that provides a warning sign once the urine has been detected by the detector [3]. Compliance of the child and his parents, besides the motivation of the child, determines the eventual success of the treatment [6].

Alarm therapy is considered the first treatment modality of choice for enuresis; cure rates are almost 50% in the long term [7]. In many studies, alarm therapy showed to be very effective to attain dryness, providing a better treatment response and a lower recurrence rate as compared to other modalities of treatment [3,8-13].

Training the kid to wake up for micturition before incontinence or to prevent emptying the bladder while asleep is the goal of alarm therapy [7]. However, waking up for micturition is not the only effect of alarm therapy. Bladder capacity has been found to be increased at night with the use of alarm therapy in many studies [14].

After alarming, if there was a failure to awaken spontaneously by the children, their parents should wake them up to void the bladder on the toilet. If both (children and their parents) didn’t wake, which occurs frequently, making a code word agreed upon previously between the parents and the child can be advised to help in waking up [4].

Ten to 12 weeks after the treatment, success rates are observed to be 50% and 80% while 12%-30% of cases can have a relapse in the first six months after treatment. However, a second course of alarm therapy can give a good response if a relapse occurs [7].

Alarm types

In many studies, four forms of night alarms were observed: sound, vibration, one that mixes an electrical impulse and a sound, and code words. The one that provides a sound is the most frequently used alarm. This type of alarm has been tested with various intensities and situations such as being associated with light or not, awakening the child immediately or after three minutes, putting a moisture sensor within the child’s underclothing or just on the bed, and finally alarming the parents or the kids only. However, there is no significant difference in terms of efficacy across the different forms of alarms [3].

Optimal duration

If alarm therapy has been decided, the duration of the therapy should be two to three months or until being dry for 14 consecutive days. However, the most effective period of alarm intervention is anticipated to be 16-20 weeks of continuous therapy. Sleep deprivation can result due to repeated alarm, so one alarm per night is enough. Despite its high efficacy, choosing alarm treatment is critical because it is not easy to find the right families for this option and to inform them in a comprehensive manner, as the possibility of frustration and withdrawal among parents and children is not uncommon [4,13,15].

Discussion

There are multiple factors that might slow down the therapeutic response time. One of these factors is the technical issues with the alarm such as the child's sweat or the battery not lasting long enough. Some devices only have a moisture detector, which might cause issues when trying to activate the alarm after an enuresis episode. Another aspect is the kind of fabric used for the moisture detector, requiring higher or lower absorption rates, as well as the alarm's technical quality, which can affect therapeutic efficacy [3].

## Conclusions

To conclude, alarms are still the preferred and most effective treatment for enuresis. Regarding alarm types, there is no consensus that there is a significant difference between the alarm types in terms of effectiveness. Cooperation and compliance from parents and children are the cornerstones of the efficacy of alarm therapy. Several factors may impede the therapeutic period of this treatment, which should be taken into account. Larger and further studies are needed to avoid these issues.
